# Promoting workplace psychological wellbeing: evaluation of a multidisciplinary Employee Assistance Program at a tertiary hospital in Asia

**DOI:** 10.3389/fpubh.2025.1711622

**Published:** 2026-01-27

**Authors:** Kenneth Bao Ren Leong, Sharon Shujin Tan, Say Leong Ooi, Wen Phei Lim, Katie Kai Teng Lim, Nur Syafilla Iqma Samat, Hui Zhu, Asanachiyaar Chinnathamby, Rosman Bin Surie, Jeremiah Chng, Jeff Yi-Fu Hwang

**Affiliations:** 1 Preventive Health and Occupational Medicine, Woodlands Hospital, Singapore, Singapore; 2 HR Centre of Excellence, Woodlands Hospital, Singapore, Singapore; 3 Department of Psychology, Woodlands Hospital, Singapore, Singapore; 4 Medical Psychiatry, Woodlands Hospital, Singapore, Singapore; 5 Ambulatory Care (SOC), Woodlands Hospital, Singapore, Singapore

**Keywords:** Employee Assistance Program, occupational medicine, occupational mental health, psychiatry, psychological wellbeing, psychology

## Abstract

**Background:**

Despite the growing availability of Employee Assistance Programs (EAP) aimed at improving workplace psychological wellbeing, the implementation and effectiveness of EAPs has not been well described nor well studied in the literature. This study seeks to describe and evaluate an insourced, multidisciplinary EAP consisting of Occupational Medicine Physicians, Psychiatrists, Psychologists as well as Human Resource Professionals to promote psychological wellbeing among healthcare workers in a tertiary hospital in Singapore.

**Methods:**

This study utilized a health service evaluation framework and analyzed the implementation of the EAP across five dimensions, namely: Reach and Adoption, Effectiveness, Implementation and Maintenance. Anonymous longitudinal data of all participants enrolled into the EAP program between 01 Jan 2024 to 30 April 2025 were collected for analysis.

**Results:**

Data from a total of 39 EAP participants were analyzed. Nursing staff formed the largest proportion of staff who utilized the EAP at 51.3%. The most common route of access to the EAP program was through referral by the staff’s department at 43.6%, followed by self-referral (23.1%) and referral by a peer-supporter (23.1%). The most common reason for EAP attendance was work-related stressors at 48.7%. A statistically significant decrease between the median pre-EAP Patient Health Questionnaire-4 (PHQ-4) score (7) and median post-EAP PHQ-4 score (2) was noted. 59% of participants were able to return to work. An estimated average running cost of $648.48 per participating staff was required to sustain the program.

**Conclusion:**

This is the first longitudinal study in Southeast Asia describing the evaluation of an EAP. Using an objective clinical questionnaire, an improvement in psychosocial wellbeing was noted for EAP participants. The evaluation methods and outcomes described provide a framework for companies and human resources department to review ongoing EAPs as the organization and structure of EAPs continue to evolve.

## Introduction

As industries and workplaces continue to evolve, there has been an increasing emphasis placed upon addressing psychological wellbeing at the workplace. Compared to the previous decade, work practices have evolved as a result of increasing digitalisation, use of artificial intelligence as well as the COVID-19 pandemic. These ongoing changes in the working environment can bring various psychological stressors that may negatively affect an individual’s psychological wellbeing.

In the healthcare workplace, the psychological stressors that a healthcare worker faces are manifold and complex, ranging from cumulative stress to isolated traumatic incidents. This was amplified during the COVID-19 pandemic with many studies finding a high prevalence of burnout among healthcare providers. In Singapore, a study conducted during the COVID-19 pandemic showed that more than one in three healthcare providers suffered from burnout (1). Despite facing these psychological stressors, healthcare providers may face barriers in seeking help given the potential stigma and risk of losing their professional licensure as a result of declaring mental health issues. Such delays in seeking help may result in impaired staff performance, lower staff satisfaction and ultimately staff attrition.

One common impetus for promoting good psychological wellbeing at the workplace has been to protect productivity (2). Based on the Singapore Mental Health Study, persons with poor mental health experience 0.5 days per month of absenteeism, resulting in an annualized national projection of approximately 0.3 million productivity days lost as a result of mental health conditions (3). Given the organizational impacts of poor psychological wellbeing, there is a strong need to cultivate psychological wellbeing among healthcare workers.

Apart from the organizational perspective, there is also a growing body of evidence that good psychological wellbeing is associated with improved physical and mental health (4) for the individual. Research has shown that individuals experiencing higher levels of optimism were more likely to engage in healthy lifestyles, such as exercise, and cut down on unhealthy behaviors, such as smoking (5–7). Conversely, poor psychological wellbeing has been linked with the development of mental health disorders, such as anxiety and depression.

As a result of the increased focus on psychological wellbeing, companies across the world have progressively implemented programs to improve workplace psychological wellbeing. The Employee Assistance Program (EAP), which can be described as a work-based program that encompasses a wide spectrum of services to address aspects of work, life, and health (8), is an example of a program that has seen widespread adoption by companies across many countries. Locally in Singapore, the experience has been similar. In 2017, a Singapore study found that less than 5% of employers offered EAP programs for their staff (9). However, since the release of the Tripartite Advisory on Mental Well-being at Workplaces by the Ministry of Manpower, awareness and understanding of mental health concerns at workplaces has been growing.

EAP services can be offered within different organizations by a diverse array of professionals such as human resource professionals, psychologists, psychiatrists and physicians. There is also a diverse array of interventions such as workplace mediation, counseling, supervisor mentoring, and trauma training. Additionally, there is also significant variability in the implementation of EAP programs, with certain companies opting for completely insourced services while other companies would procure outsourced services.

As there are many factors affecting one’s emotional and mental health, multidisciplinary interventions involving a mix of health professionals could complement Human Resource Professionals in improving the outcomes for staff enrolled in an EAP. For example, an EAP which involves Occupational Medicine (OM) Physicians could be beneficial as OM physicians are able to use their understanding of workplace exposures and processes to assist workers in coping with psychological stressors and to assess the worker’s fitness for work. Psychiatrists and Psychologists can also contribute by providing ready access to psychological health services, such as counseling, behavioral therapy as well as pharmaceutical treatments. As such, a hybrid model comprising of insourced and outsourced services comprising of interventions by various professional groups (Occupational Medicine, Psychiatry, Psychologists, HR professionals) was implemented in a tertiary hospital in Singapore to promote psychological wellbeing.

To the best of our knowledge, the implementation of such multidisciplinary initiatives to improve workplace psychological wellbeing has not been well described nor well studied in the literature (8). Additionally, there is also a lack of studies investigating the impact of EAPs on psychological wellbeing (10). Evaluating the effectiveness of EAPs is important given that companies invest substantial resources into such programs and that there are considerable differences in the mode of implementation as well as the scope and activities offered within the EAP. As such, this study seeks to describe and evaluate an insourced, multidisciplinary EAP consisting of Occupational Medicine Physicians, Psychiatrists, Psychologists as well as Human Resource Professionals to promote psychological wellbeing among healthcare workers in a tertiary hospital in Singapore. Findings from this study will provide evidence on the effectiveness and impact of multi-disciplinary EAPs and help to guide quality improvement initiatives for EAP programs.

## Methods

### Implementation of the Employee Assistance Program

Woodlands Hospital (WH) is a tertiary hospital in Singapore which provides a comprehensive range of acute, sub-acute, rehabilitative and transitional care services for patients and has up to 1,000 beds in the acute and community hospital and up to 400 beds for long term care patients. As of 2024, the hospital has a staff strength of over 3,000 healthcare workers supporting the operations of the hospital.

In December 2023, WH initiated a multidisciplinary psychological wellbeing program, known as the Woodlands Hospital Employee Assistance Program (WH EAP) to support its healthcare workers with psychological difficulties at work. This includes staff who have experienced abuse or harassment at work or other forms of work-related distress, such as being second victims of adverse medical incidents.

**Figure 1:**
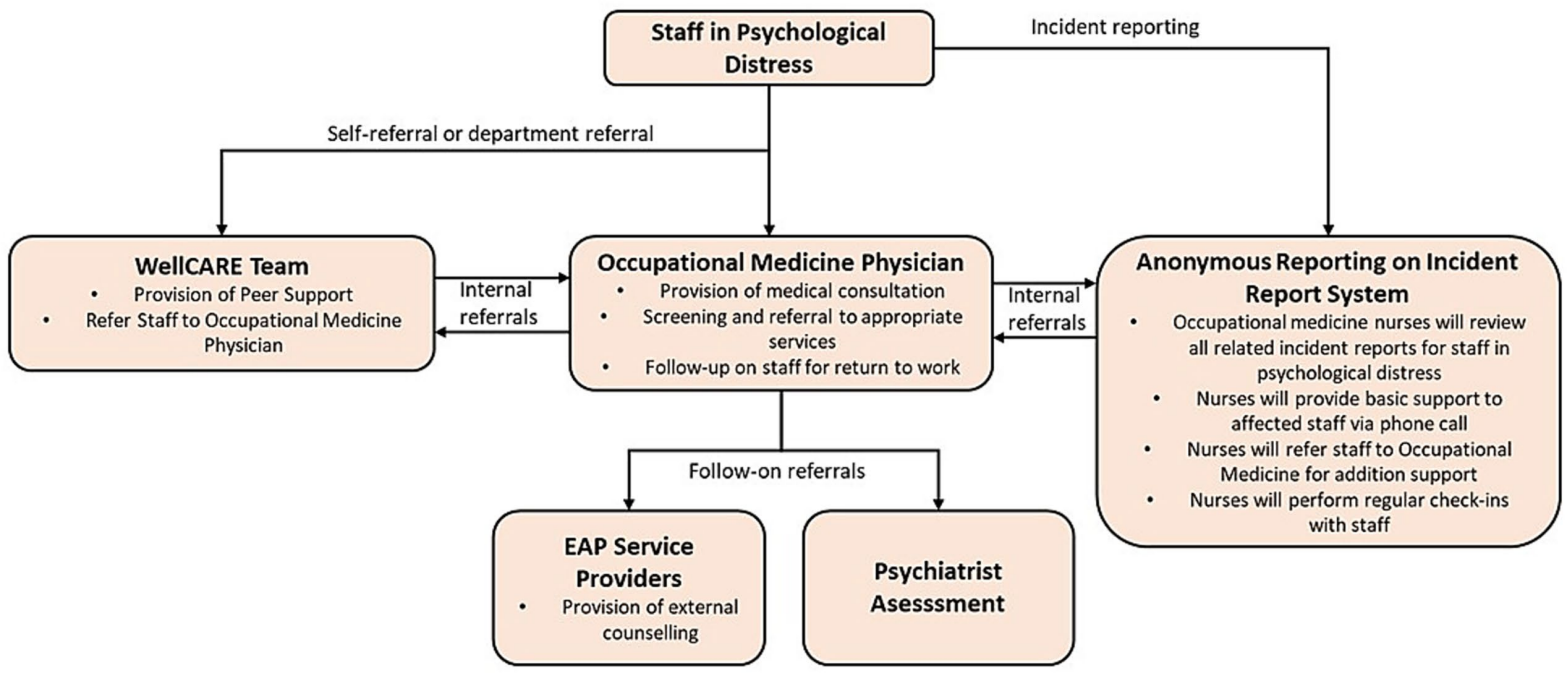
Woodlands Hospital Employee Assistance Program overview.

WH EAP is a multidisciplinary initiative comprising of Occupational Medicine Physicians, Nurses, Psychiatrists, Psychologists and Human Resource Professionals. An overview of the program is summarized in Figure 1. Staff who experience psychological distress can walk-in to the Occupational Medicine clinic or be referred by their supervisor or peers (i.e., WellCARE) to access the WH EAP service. In addition, staff who file incident reports in the Occupational Health and Safety Management System (PRISM) and have incidents related to psychological distress will be contacted and offered the EAP service. WH EAP offers an initial assessment by occupational medicine doctors and nurses to any staff who present with psychological distress. The function of the physician or the nurse would be to provide initial medical assessment to assess the staff’s symptoms to triage and refer the staff to appropriate services. The medical assessment includes assessment of suicide risk, clinical depression and anxiety through history taking, physical examination and use of validated psychometric assessment scales.

Acutely suicidal staff will be escorted to the emergency department for urgent psychiatric assessment and treatment. For non-urgent cases, the occupational physician may consider prescribing symptomatic treatment with outpatient follow-up and either outpatient referrals to the psychiatrist, external counseling services or peer supporters. The occupational physician provides closed-loop care by following up with the staff to assess their symptoms and fitness to return to work even after they have been referred to the psychiatrist or external counselor. If required, monthly multidisciplinary case discussion meetings may be convened to discuss the management of cases using anonymised case information. Furthermore, with the consent of the staff, Occupational Medicine specialists can also provide recommendations to HR professionals and supervisors in order to facilitate return-to-work arrangements. By providing such closed-loop care for staff, the EAP is able to play a greater role in facilitating the staff’s eventual return to work by working with the staff on return-to-work arrangements and assisting with light duty, medical leave arrangements or job design.

### Evaluation framework

This study utilized a health service evaluation framework to evaluate an insourced, multidisciplinary Employee Assistance Program consisting of Occupational Medicine Physicians, Psychiatrists, Psychologists, Nurses as well as Human Resource Professionals to promote psychological wellbeing among healthcare workers in a tertiary hospital in Singapore.

The RE-AIM framework, one of the most frequently applied implementation frameworks (11), was utilized to assess five dimensions: reach, effectiveness, adoption, implementation and maintenance. Using the RE-AIM framework, the study team evaluated the multidisciplinary program in the five dimensions as described below.

#### Reach and adoption

These two dimensions assesses the number and characteristics of individuals who participated in the program. To evaluate reach, demographic data was collected regarding the participants. In addition, data was also collected on how many individuals participated as well as their job group and departments to evaluate the proportion and representativeness of settings (e.g. departments within the hospital) that benefitted from the EAP.

#### Effectiveness

This dimension measures the impact of the initiative on psychological wellbeing outcomes. Post-intervention improvements were analyzed using validated psychological scales (e.g. PHQ-4) to determine changes in mental health status.

#### Implementation

This assesses the quality of the program delivery. Data collected to evaluate this dimension include the number of consultations sessions held and accessibility of the program.

#### Maintenance

This dimension looks at the long-term sustainability of the initiative. Data on resource allocation and cost effectiveness were analyzed to provide insights into the program’s sustainability.

### Data sources

The data source comprised of anonymised case information extracted from an operational database used for clinical care delivery as well as an anonymous survey collecting feedback on participant experience for the purposes of quality improvement. Total population sampling of all participants in the EAP was conducted in view of the small study population. As such, data from all participants of the EAP were included for analysis. Data of participants were collected for those who were enrolled into the WH EAP program between 01 Jan 2024 to 30 April 2025.

### Ethical approval

The research proposal was presented to the National Healthcare Group Institutional Review Board (CIRB) and exempted from review as the study was part of a quality improvement and review process to improve the psychological wellbeing of healthcare staff. No directly identifiable personal data was collected and only aggregate data was reported. Data collected in the course of the study was stored securely on an encrypted repository.

### Statistical analysis

All results were analyzed using STATA version 13 (Stata Corporation, TX). To evaluate the Reach and Adoption of the program, descriptive statistics will be presented to evaluate the ease of access as well as employee groups who benefitted from the program. To evaluate the effectiveness of the program, patient reported outcome measures, such as the Patient Health Questionnaire-4 (PHQ-4) as well as return-to-work status was examined. Tests for statistical significance, such as Wilcoxon Signed-Rank Test was used to evaluate the efficacy of the program in various subgroups based on demographic and work-related factors. Confidence intervals for return-to-work status was also estimated by assuming a binomial distribution.

## Evaluation results

### Reach and adoption

Over the course of January 2024 to April 2025, a total of 39 staff were seen under the Employee Assistance Program. Two thirds of the staff seen were female. 82% of the staff belonged in the younger age group (<40 years old). 41% of staff who utilized the EAP were foreigners. Nursing staff formed the largest proportion of staff who utilized the EAP at 51.3%, followed by Allied Health and Pharmacy at 20.5%. Medical staff had the lowest proportion at 2.6% (Table 1).

**Table 1:** Demographics of participants in WH EAP (*N* = 39).

Demographic variables	*n* (%)
Gender	
Female	26 (66.7)
Male	13 (33.3)
Age (years)	
20–29	19 (48.7)
30–39	13 (33.3)
40–49	5 (12.8)
50–59	2 (5.1)
Nationality	
Singaporean/PR	23 (59.0)
Foreign	16 (41.0)
Referral source	
Self-referred	9 (23.1)
Referred by Department	17 (43.6)
Referred by Occ Med Nurse through PRISM reporting	4 (10.3)
Referred by WellCARE	9 (23.1)
Family group	
Nursing	20 (51.3)
Admin	7 (17.9)
Allied Health / Pharmacy	8 (20.5)
Ancillary	3 (7.7)
Medical	1 (2.6)
Psychological stressor	
Abuse by patient	6 (15.4)
Work stressor	19 (48.7)
Non-work stressor	8 (20.5)
Both work and non-work stressors	6 (15.4)

The most common route of access to the WH EAP program was through referral by the staff’s department at 43.6%, followed by self-referral (23.1%) and referral by a peer-supporter (i.e. WellCARE) (23.1%) (Table 1).

In terms of reasons for attending the WH EAP, the most common reason was work-related stressors at 48.7%. Notably, 20.5% of participants attended the WH EAP due to non-work stressors. 15.4% of participants attended due to both work and non-work stressors and another 15.4% of participants attended due to recent abuse or harassment by patients (Table 1).

In terms of accessibility of the EAP service, staff who encountered patient abuse received OM consultations comparatively earlier (Table 2) with a median time of 0.9 weeks, compared to staff facing other forms of psychological distress. Staff facing mainly work stressors took the longest time to present, with a median time of 10.9 weeks.

**Table 2:** Time from incident or initial symptom to OM consult, by main psychological stressor.

Main psychological stressor	Time in weeks, median (IQR)
Abuse by patient (*n* = 6)	0.9 (0.4)
Work stressor (*n* = 19)	10.9 (10.6)
Non-work stressor (*n* = 8)	8.4 (9.1)
Both work and non-work stressors (*n* = 6)	8.9 (9.3)

### Effectiveness

#### PHQ-4 score

A total of 25 out of 39 participants with PHQ-4 scores recorded during the initial consult and post EAP program were analyzed. Post EAP PHQ-4 scores were measured during the last consultation prior to discharge from the EAP. 4 participants were still on follow-up and hence the post EAP PHQ scores were not available. 10 participants did not require follow-up after the first visit so no further PHQ scores were collected. Reasons for not requiring follow-up include participants leaving the organization, participants who declined further follow-up as well as those presenting with mild symptoms that were discharged from the program after the first visit.

In the entire cohort, an overall statistically significant decrease in PHQ-4 scores was noted when the initial median EAP PHQ-4 scores (7) were compared against the Post EAP median PHQ-4 scores (2). There was a decrease in PHQ-4 scores across all demographic subgroup factors such as Gender, Age and Nationality. PHQ-4 scores also decreased across all job groups, except for Medical. In subgroup analysis by referral source, the initial and post PHQ scores for participants who were enrolled due to screening of PRISM reports remained the same (Table 3).

**Table 3:** Pre and post PHQ-4 scores.

Demographic variables	Initial median PHQ-4 score (IQR)	Post EAP median PHQ-4 score (IQR)	*p*-value
Overall (*n* = 25)	7 (3–10)	2 (0–5)	<0.01*
Gender			
Female (*n* = 19)	5 (3–9)	3 (1–5)	<0.01*
Male (*n* = 6)	9.5 (6–12)	1 (0–2)	0.03
Age			
20–29 (*n* = 12)	5.5 (2.5–9)	1 (0–4)	<0.01*
30–39 (*n* = 8)	10 (6.5–12)	3 (1.5–6)	0.02*
> = 40 (*n* = 5)	5 (3–7)	3 (2–4)	0.16
Nationality			
Singaporean/PR (*n* = 14)	6 (5–10)	2.5 (0–5)	<0.01*
Foreigner (*n* = 11)	8 (2–10)	2 (0–5)	0.03*
Family group			
Nursing (*n* = 15)	8 (3–10)	2 (0–5)	<0.01*
Allied Health and Pharmacy (*n* = 4)	6.5 (4–8)	3.5 (0.5–7)	0.10
Admin (*n* = 2)	5 (5–5)	2.5 (1–4)	0.17
Ancillary (*n* = 3)	11 (3–12)	3 (0–4)	0.17
Medical (*n* = 1)	2 (N. A.)	5 (N. A.)	0.31
Referral Source			
Self-referred (*n* = 6)	8 (5–8)	4.5 (3–6)	0.05*
Referred by Department (*n* = 10)	8.5 (5–12)	2 (0–4)	<0.01*
Referred by Occ Med Nurse through PRISM reporting (*n* = 3)	3 (2–3)	2 (0–6)	1.00
Referred by WellCARE (*n* = 6)	6.5 (2–10)	0.5 (0–2)	0.07
Psychological stressor			
Abuse by patient (*n* = 4)	3 (2.5–6.5)	1 (0–4)	0.45
Work stressor (*n* = 14)	5.5 (3–8)	2 (0–5)	<0.01*
Non-work stressor (*n* = 3)	12 (9–12)	6 (4–8)	0.11
Both work and non-work stressors (*n* = 4)	9.5 (6.5–11)	1 (0.5–2)	0.07
Return to work status			
Retained (*n* = 15)	5 (3–10)	1 (0–3)	<0.01*
Resigned (*n* = 10)	8 (5–10)	4.5 (2–6)	0.03*

*Statistically significant.

#### Return to work status

Return to work status was examined at a time period of 6 months following the initial consultation. Return to work was defined as resuming full work duties without specialized job restrictions or light duties. A total of 22 out of 39 participants return to work status was collected. The return to work status of the remaining 17 participants was not collected as the initial consultation was still within the last 6 months at the time of data collection and analysis. Among the study cohort, 59% of participants were able to return to work (Table 4).

**Table 4:** Return to work status 6 month post initial presentation.

Demographic variables	Percentage of participants returned to work (*n*)	95% CI
Overall	59.1% (13)	36.4–79.3
Gender		
Female	50.0% (7)	23.0–77.0
Male	25.0% (2)	3.2–65.1
Age		
20–29	36.4% (4)	10.9–69.2
30–39	40.0% (2)	5.3–85.3
> = 40	50.0% (3)	11.8–88.2
Nationality		
Singaporean/PR	46.2% (6)	19.2–74.9
Foreigner	33.3% (3)	7.5–70.1
Family group		
Nursing	58.3% (7)	27.7–84.8
Allied Health and Pharmacy	25.0% (1)	1.0–80.6
Admin	100% (3)	29.2–100.0
Ancillary	100% (2)	15.8–100.0
Medical	0% (0)	0.0–97.5
Referral source		
Self-referred	25.0% (1)	1.0–80.6
Referred by Department	50.0% (5)	18.7–81.3
Referred by Occ Med Nurse through PRISM reporting	100.0% (2)	15.8–100.0
Referred by WellCARE	83.3% (5)	35.9–99.6
Psychological stressor		
Abuse by patient	100% (4)	39.8–100.0
Both work and non-work stressors	100% (5)	47.8–100.0
Non-work stressor	0% (0)	0.0–60.2
Work stressor	36.4% (4)	10.9–69.2

In subgroup analysis, there was a decrease in EAP scores in both groups of participants who eventually resigned compared to participants who remained in employment. However, participants who resigned had a higher initial (8 vs. 5) and post EAP PHQ-4 score (4.5 vs. 1) as compared to participants who returned to work (Table 3).

Stratified by referral source, return to work status was lowest among those who were self-referred at 25% and highest among those who were enrolled due to screening of PRISM reporting at 100%. Stratified by psychological stressor, return to work status was lowest among staff who were seen for non-work stressors at 0% (Table 4).

### Implementation and maintenance

A total of 63 occupational medicine consultations and 44 external counseling sessions were performed for a total of 25 discharged patients who presented in the period of January 2024 to April 2025.

Each staff received an average of 2.52 occupational medicine consultations and 1.75 sessions of external counseling in the WH EAP program. There were no statistically significant differences in the number of occupational medicine consultations or external counseling sessions based on demographic factors as shown in Table 5.

**Table 5:** Utilization of Employee Assistance Program.

Demographic variables	Average number of occupational medicine consults per staff (SD)	*p* value	Average number of external counseling sessions per staff	*p* value
Overall (*n* = 25)	2.52 (1.48)	N.A.	1.75 (1.75)	N.A.
Gender				
Female (*n* = 17)	2.24 (1.09)	0.16	1.75 (1.65)	1.00
Male (*n* = 8)	3.13 (2.03)		1.75 (2.05)	
Age				
20–29 (*n* = 12)	2.75 (1.91)	0.48	1.91 (1.92)	0.58
30–39 (*n* = 8)	2.63 (1.06)		2.00 (1.85)	
> = 40 (*n* = 5)	1.80 (0.44)		1.00 (1.22)	
Nationality				
Singaporean/PR (*n* = 15)	2.60 (1.55)	0.75	1.79 (1.72)	0.90
Foreigner (*n* = 10)	2.40 (1.43)		1.70 (1.89)	
Family group				
Nursing (*n* = 13)	2.85 (1.82)	0.75	1.62 (1.76)	0.64
Allied Health and Pharmacy (*n* = 3)	2.33 (0.58)		2.33 (1.15)	
Admin (*n* = 7)	1.86 (0.90)		1.33 (1.86)	
Ancillary (*n* = 2)	3.00 (1.41)		3.00 (2.83)	
Referral source				
Self-referred (*n* = 3)	3.00 (0.82)	0.70	1.75 (0.96)	0.62
Referred by Department (*n* = 12)	2.67 (1.92)		1.64 (1.86)	
Referred by Occ Med Nurse through PRISM reporting (*n* = 4)	1.75 (0.50)		1.00 (1.41)	
Referred by WellCARE (*n* = 5)	2.40 (1.14)		2.60 (2.30)	
Psychological stressor				
Abuse by patient (*n* = 6)	1.67 (0.52)	0.36	1.00 (1.26)	0.17
Both work and non-work stressors (*n* = 6)	3.17 (1.47)		3.00 (2.28)	
Non-work stressor (*n* = 3)	2.33 (1.53)		1.00 (0)	
Work stressor (*n* = 10)	2.70 (1.77)		1.60 (1.58)	

## Discussion

### EAP outcomes

Current evidence regarding the effectiveness of EAPs in the region have been limited to cross-sectional questionnaire-based studies (12). Our study expands upon the existing literature by analyzing the longitudinal outcomes among a cohort of staff who face psychological stressors. In addition, our study captured both productivity related outcomes, such as return to work status, as well as clinical outcomes in the form of the PHQ-4 score. The PHQ-4 (Patient Health Questionnaire-4) is a brief screening tool used in clinical practice to assess the presence of anxiety and depressive symptoms (13). It consists of four questions, with two questions related to depression and two related to anxiety. In the context of this study, the PHQ-4 scores of participants during the initial occupational medicine consultation and at discharge were collected for two purposes. Firstly, the scores were used as an indicator for the effectiveness of the EAP program. Secondly, the scores were also used to evaluate the severity of the psychosocial stressors faced by the healthcare worker.

As evidenced from the improvement of the PHQ-4 scores, the EAP program can be deemed to be effective in addressing psychosocial stressors which has been reflected as a decrease in the PHQ-4 scores from the initial consultation until the point of discharge. Interestingly, participants who resigned had a higher initial and higher post EAP PHQ-4 score as compared to participants who returned to work. These results may indicate that staff who experienced a higher degree of psychological stressors would be at higher risk of not being able to return to work. Given the possible association between PHQ-4 scores and return to work status, this study highlights the importance of incorporating clinical indicators such as PHQ scores in EAP programs, thus allowing for the effectiveness of such programs to be evaluated with a greater level of detail beyond participant satisfaction and attrition.

In terms of the severity of depressive and anxiety symptoms faced by healthcare staff, the results indicate that staff with non-work stressors faced more severe symptoms, as evidenced by a higher PHQ-4 score, as compared to staff who faced work-related stressors. This highlights the high burden of non-work related stressors that healthcare workers might face which may be as severe or even more severe when compared to work-related stressors. In previous studies, examples of non-work related stressors include family stressors, personal health stressors, housing stressors and inter-personal relationship stressors (14).

Staff who were referred by their department for the EAP had a higher initial PHQ-4 score as compared to staff that were self-referred. Despite having a higher initial PHQ-4 score, these staff ended up having a lower post-EAP PHQ-4 score, indicating a greater improvement in their psychological wellbeing as compared to staff who were self-referred. Correspondingly, a higher proportion of staff (i.e. 50%) who were referred by their department managed to return to work as compared to 25% of staff who were self-referred. This could point toward the importance of having a supportive department in the overall management of staff experiencing psychological stressors as it could be postulated that departments which made an effort to recommend staff for the EAP might place a higher emphasis on psychological wellbeing and would assist the affected staff in coping with the various psychological stressors.

While the PHQ-4 score has been helpful to measure workplace psychological well-being, it is also important to acknowledge its limitations. As the PHQ-4 primarily measures only anxiety and depressive symptoms, it does not provide a holistic assessment of wellbeing and productivity because staff with workplace distress may also present with symptoms of burnout. Comprehensive scales like the Maslach Burnout Inventory can provide a comprehensive assessment of burnout-related symptoms. Despite this, a calculated decision was made by the EAP team to use the PHQ-4 given its ease of use and efficiency in a fast-paced clinic setting while acknowledging that the PHQ-4 may miss nuanced aspects of burnout.

### EAP utilization and access

Apart from highlighting the longitudinal outcomes for staff facing psychological stressors, this study also sheds light on the utilization patterns that organizations with EAPs may experience. These findings would be helpful in resource planning and quality improvement. While the provision of EAPs in Singapore has been growing steadily, data regarding actual utilization has been lacking. In this study, we found the utilization of EAPs to generally be low. Over the course of 14 months, 39 staff participated in the program. This translates to an approximate of 3 staff members per month which translate to 0.1% of the total staff strength per month. Nevertheless, as shown in Table 1, the EAP served staff from a diverse range of demographic backgrounds and catered to both genders, young and old as well as staff from various nationalities and job functions. While the most common reason for EAP utilization was for work-related stressors, it is also important to provide support for staff with non-work related stressors who may also face high levels of psychological distress.

In this evaluation, staff who suffered from cases of patient abuse or harassment were promptly enrolled into the program, with a median time of 0.9 weeks between incident to enrolment. This could be partly attributed to the increased awareness and emphasis placed upon protecting healthcare workers from patient abuse. However, we also found that the median time between experiencing *non-abuse* work-related psychological stressors to seeking help at the EAP to be 10.9 weeks (Table 2). This could be postulated to be contributed by local beliefs and culture. In Singapore, there is a strong emphasis on self-reliance and a stigma associated with seeking help for mental health issues. As such, workers perceive using EAP services as a sign of psychological weakness, leading to lower participation rates. Addressing these cultural perceptions and promoting the benefits of EAPs in a culturally sensitive manner could enhance their utilization among employees in the local context.

### EAP implementation and maintenance

While the overall utilization of EAP locally may not be high, substantial effort was required in the establishment of the service given our hybrid model with a substantial number of services being insourced. Apart from catering for the additional workload, financial resources had to be obtained in order to fund the external counseling services. An estimated average running cost of USD $$479.64 per participating staff was derived based on the assumptions set out in Table 6, with costs reflected in USD according to the average monthly USD to SGD monthly exchange rate (15). In addition to these running costs, it is important to also consider other costs which may not be easily quantified. Examples of such costs include multidisciplinary case discussions involving human resource professionals, psychologists, occupational medicine physicians, nurses and psychiatrists and overall program coordination and administration. Despite the high costs of maintaining the EAP service, this needs to be balanced against the hidden costs attributed to staff turnover. While there are no local studies estimating the cost of a single staff turnover, a systematic review of costs associated with nursing turnover estimated costs ranging from around USD $21,514 to $88,000 (16). Given such high costs of nursing turnover, even a single attrition prevented would justify the running costs of an Employee Assistance Program.

**Table 6:** Estimated cost of EAP provision.

Cost category	Cost
Occupational Medicine Consultation (USD $128.70 per consultation by Consultant Rank Physician) (17)	$128.70 × 2.52* = $324.32 (*average number of consultations)
External counseling (USD 88.76 per counseling session) (18)	$88.76 × 1.75* = $155.33 (*average number of external counseling services)
Total cost	USD $479.64

### Strengths and limitations

Our study had several notable strengths. This is the first such study in Southeast Asia and one of the few in the world describing the evaluation of a multidisciplinary EAP for healthcare workers. Additionally, the incorporation of validated psychological measurements distinguishes our study from existing literature which had been focused on analyzing EAP effectiveness from a productivity and business perspective.

Nonetheless, our study had several limitations that should be acknowledged. Firstly, it was conducted at a single site, which may restrict the generalizability of the findings. The overall study size remained relatively small, potentially constraining the statistical robustness of the outcomes. These constraints were largely due to the novel structure of our WH EAP which has not been implemented before in the local context and the limited utilization of EAP due to underlying socio-cultural beliefs and practices. Future studies could investigate the effectiveness of similar EAP programs.

## Conclusion

This study provided insights on the effectiveness of a multidisciplinary EAP program for healthcare workers and evaluated the program using objective indicators of psychological health. The advantages of involving a multidisciplinary team in an EAP were also highlighted and discussed which would be useful information for companies to consider as they review their EAPs. The evaluation methods described here also provide a framework for companies and human resources department to review their EAPs as the organization and structure of EAPs continue to evolve.

## Data Availability

The raw data supporting the conclusions of this article will be made available by the authors upon reasonable request.
